# The Anatomy and Philosophy of Expression as Connected with the Fine Arts

**Published:** 1845-03

**Authors:** 


*The Anatomy and Philosophy of Expression as connected with the Fine Arts.* By Sir Charles Bell, K. H. *Third Edition Enlarged.* Royal 8vo. pp. 265. London, 1844.

These Essays—it appears from the Preface—formed the earliest and the latest occupation of the lamented Author's leisure hours. Their beauty, not only of expression, but of illustration—for Sir Charles was an accomplished limner—struck us forcibly at an early period of our professional career, and we hastened to possess ourselves of this—what may be regarded posthumous—production; for although two editions have been exhausted, the present has been prepared since his decease, with the addition of notes left by him.

The number and beauty of the illustrations render the volume necessarily expensive; but it is well worth the attention of the physician, the philosopher and the artist.

The Essays areexceedingly interesting, and comprise a comparison of ancient and modern art; the theory of beauty in the countenance; the sources of expression in the countenance; the expression of passion in man and animals; the study of anatomy as necessary to design; the uses of anatomy to the painter, &c.

An appendix on the nervous system, to the knowledge of which Sir Charles Bell made such important contributions, has been added by Mr. Alexander Shaw, the brother of Mr. Shaw, the anatomist, who died at an early age, and who was a connexion of Sir Charles. In this, Mr. Shaw gives a brief account of the leading parts of the discoveries made by Sir Charles, from which we extract the following concluding observations.

"He first established, on undoubted evidence, the fact, that the nerves of motion are distinct from those of sensation; and that the nerves generally possess different endowments, according to the divisions of the brain from which they arise. He then arranged the nerves of the whole body into three distinct systems, corresponding with the organs which they respectively control. The first class was that composed of the spinal nerves and fifth nerve of the brain; this class, he proved, bestows both motion and sensation on all the parts to which it is distributed; and these parts, he further showed, are organs which belong to man in common with the lowest creatures, their united function being to supply food, the first necessary want of all animals: he termed this set of nerves the "original" class, and included in it the various organs of the senses. The second class comprised in it a series of nerves distinct from the former, both in their origin and mode of distribution; they pass off from a circumscribed central portion of the nervous system, the medulla oblongata, and diverge to different parts of the head, neck, throat and chest, already supplied by the original class: he showed, that these structures form together the organs of respiration—a mechanism, which does not belong to the lowest animals, but is gradually introduced by a slow process of development into the animal kingdom, in order that, besides oxygenating the blood, it may become, in man, the organ of Voice and Expression: to this set of organs he applied the name respiratory class. In these two classes were combined all the nerves together, which arise either from the brain or spinal marrow. The third class consisted of a series of nerves which have their centre in large ganglions, scattered principally among the viscera of the abdomen. This forms the system called ganglionic or sympathetic; and their use has been generally supposed to be, to unite in sympathy those organs by which the various organic functions are performed; such as secretion, absorption, assimilation of the food, the growth and decay of the body, &c. When the nerves belonging to these different classes are viewed in their combined condition, as seen by the anatomist, nothing can exceed their apparent confusion; but when examined by the aid of the principle, and the arrangement introduced by Sir Charles Bell, order and design are found to pervade every part."

We would not detract one tittle from the well-earned reputation of Sir Charles Bell; yet it must be admitted, that his views on the respiratory system of nerves have not stood the test of subsequent examination. There does not, indeed, seem to be in the spinal marrow any distinct tract or column of respiration such as he depicted; although, doubtless the nerves designated by him are largely concerned with respiration and expression; moreover, certain of these nerves—as the pneumogastric—belong to the true spinal system of nerves, or to the gray matter of the associated ganglia that form the centre of the spinal marrow. Mr. Shaw has not alluded to the important addition to our knowledge of the nervous system made of late years, through the labours of Dr. Marshall Hall more especially, by which we are enabled to explain so many healthy and morbid phenomena, previously unintelligible to us. The determination of the distinct functions of the two roots of the spinal nerves belongs to Sir

Charles Bell; and on it his future fame will rest more than on all his other labours. His reputation as a surgeon is altogether merged in that of the anatomist and physiologist.

